# Mapping the genetic and clinical characteristics of Gaucher disease in the Iberian Peninsula

**DOI:** 10.1186/1750-1172-7-17

**Published:** 2012-03-19

**Authors:** Pilar Giraldo, Pilar Alfonso, Pilar Irún, Laura Gort, Amparo Chabás, Lluïsa Vilageliu, Daniel Grinberg, Clara M Sá Miranda, Miguel Pocovi

**Affiliations:** 1 Centro de Investigación Biomédica en Red de Enfermedades Raras (CIBERER), Zaragoza, Spain; 2 Instituto de Investigación Sanitaria de Aragón (IIS), Zaragoza, Spain; 3 Spanish Gaucher Disease Foundation (FEETEG), Zaragoza, Spain; 4 Barcelona Institut de Bioquímica Clínica (Errores Congénitos del Metabolismo), Hospital Clinic; IDIBAPS, Barcelona, Spain; 5 Barcelona Departament de Genètica, Facultat de Biologia, Universitat de Barcelona; IBUB, Barcelona, Spain; 6 Portuguese Coordinating Committee for the Treatment of Lysosomal Storage Diseases, CCTDLS (1993-2005), Institute for Molecular and Cell Biology of Porto, Porto, Portugal; 7 Departamento de Bioquímica y Biología Molecular y Celular, Universidad de Zaragoza, Zaragoza, Spain; 8 S° Hematología Hospital Universitario Miguel Servet, P° Isabel La Católica, 1-3 50006 Zaragoza, Spain

**Keywords:** Gaucher disease, Glucocerebrosidase, Phenotype, Genotyping, Iberian Peninsula

## Abstract

**Background:**

Gaucher disease (GD) is due to deficiency of the glucocerebrosidase enzyme. It is panethnic, but its presentation reveals ethnicity-specific characteristics.

**Methods:**

We evaluated the distribution, and clinical and genetic characteristics of GD patients in the Iberian Peninsula (IP). We analysed geographical distribution, demographic, genetic and clinical data, age at diagnosis, type, and years of therapy in 436 GD patients from the IP.

**Results:**

The prevalence of GD was 1/149,000 inhabitants; 88.3% were type 1, 6.7% type 2, and 5.0% type 3. The mean age at diagnosis in type 1 was 28.7 years. A total of 72.7% were classified as having mild forms, 25.5% moderate, and 1.7% severe. Anemia and thrombocytopenia were present in 56% and 55%, respectively. Bone disease and hepatomegaly were reported in 62% and 68%, respectively, and were more likely in asplenic than in non-splenectomized patients. Sixty-nine mutant alleles were identified, and five mutations accounted for 75% of the *GBA *alleles. Several patients described in our series had interesting phenotypes. A total of 58.7% of patients had received enzyme replacement therapy and 12.6% were treated with miglustat.

**Conclusions:**

A broad spectrum of *GBA *mutations is present in the IP, with 98.2% of type 1 GD being mild and 23.0% never treated. These data highlight genetic and phenotypic heterogeneities among geographic populations.

## Background

Gaucher disease (GD), one of the most prevalent lysosomal storage diseases worldwide, is inherited in an autosomal recessive mode and is usually caused by deficient activity of the glucocerebrosidase enzyme (EC 3.2.1.45) [1]. The predominant clinical manifestations of the disease are cytopenias, hepatosplenomegaly and bone disease. Patients with GD are divided into three types, based on the presence and rate of progression of neurologic manifestations: type 1 non-neuronopathic (MIM 230800), type 2 acute neuronopathic (MIM 230900), and type 3 subacute neuronopathic (MIM 231000). The type 1 form is the most common among Caucasian patients but has a strong presence in the Ashkenazi Jewish population. In contrast, there is a high frequency of type 3 in Norrbotten, Northern Sweden, in Norrbotten [2]. Most Chinese GD patients have an early age of onset, severe hematological and skeletal complications, and frequent neurological involvement, resulting in early childhood death [3].

The human glucocerebrosidase gene (*GBA*) is located on chromosome 1q21 and consists of 10 introns and 11 exons. A highly homologous *GBA *pseudogene lies 16 kb downstream from the functional gene [4]. More than 300 mutations, including point mutations, deletions, insertions, splicing aberrations and various rearrangements, have been described in the *GBA *gene region as the cause of GD (http://www.hgmd.org; April 2011). A huge variation in the distribution of mutations has nevertheless been observed in different populations. The gene mutations have been divided into three groups according to their phenotypic effect: null, severe, and mild [1]. Patients carrying at least one mild mutation have non-neuronopathic disease (GD type 1), while patients carrying two severe mutations or a severe and a null mutation usually develop neurological symptoms (GD types 2 and 3). For instance, it has traditionally been considered that the N370S allele is not associated with neuronopathic disease and that the presence of one copy of this allele is associated with type 1 GD. However, several recent reports have described some neurological manifestations in compound heterozygous individuals when one mutation is N370S [5]. In contrast, homozygosity for L444P indicates that the patient is at risk of severe disease, and fetuses with two null alleles are non-viable [6,7]. Despite these observations, genotype-phenotype correlation provides guidance, but is not an absolute predictor of outcome [8].

The N370S mutation has not been found in Japanese patients, in contrast to the L444P mutation, which is the most common mutation in this ethnic group [9]. Although four mutated *GBA *alleles (N370S, L444P, c.84insG, and IVS2 + 1 g > a) account for most reported cases in the Ashkenazi Jewish population, a broad spectrum of mutations in the *GBA *gene cause GD in Caucasian patients [6].

The current Iberian Peninsula (IP) genetic pool has been influenced by many major populations and immigrations, including the Paleolithic Iberian population, which already existed by 50,000 B.C.E. Later immigrants came from North Africa, entering the Iberian region between 20,000 and 8,000 B.C.E, and the Sahara, arriving between 8,000 and 4,000 B.C.E. In addition, people arrived from central Europe (also generically called the Celtic invasions) during the first millennium B.C.E. At the beginning of the eighth century, Islamic peoples (generically called Arabs) entered Spain [10,11]. Taking into account all these data, it is believed that the timing of population divergence within the Iberian Peninsula points to a shared ancestry of all populations of the Upper Paleolithic. Further genetic subdivision is apparent in Catalonia and Andalusia, with increased genetic diversity in the latter. Lineage diversity comparisons among IP populations, European (Tuscan) and North African (Algerian) populations show that the IP is more similar to other European populations, although a small number of Iberian lineages can be traced to North Africa.

Since the early 1970s, the groups of Dr. Chabás in Barcelona (Spain) and Dr. Sa Miranda in Porto (Portugal) have focused on the identification and study of lysosomal storage diseases, including GD [12-14]. Moreover, since 1993, the Spanish Foundation for the Study and Treatment of Gaucher Disease (FEETEG) has kept the Spanish Registry of GD (SRGD) and also coordinates the screening, diagnosis, characterization, treatment, and follow-up of GD patients in Spain [15,16]. To gain greater depth of knowledge about GD and especially its clinical presentation, genotype distribution and genotype-phenotype associations, we report here the molecular characterization and associated phenotypes of a series of 436 GD patients diagnosed in the IP.

## Methods

### Data collection

The dataset for this analysis included all GD patients registered in Portugal and Spain. Data were gathered from the National Spanish Gaucher Disease Registry (since 1993), the Institut de Bioquímica Clínica, Hospital Clínic and the Genetic Department of Barcelona (in the period 1976-2002), and the Portuguese Coordinating Committee for the Treatment of Lysosomal Storage Diseases group (in the period 1993-2005). Demographic and clinical data were collected from all the referred GD patients during clinic visits. In addition, a clinical evaluation was provided by the patient's physician, and patients gave information about ethnic background. The following baseline demographic characteristics were recorded for each patient in the study: age, sex, ethnicity (provided by the patients), *GBA *genotype, country of origin, and status (alive/dead). Clinical characteristics of GD at diagnosis included: age at diagnosis; organomegaly; spleen status; cytopenias; hemoglobin, ferritin, and gammaglobulin levels; Severity Score Index (SSI) [17]; type of treatment; and years of therapy. We used the Spanish-MRI (S-MRI) to establish the degree of bone disease [18].

### Diagnosis

The diagnosis of GD was established by the demonstration of low glucocerebrosidase activity in leukocytes or fibroblasts [19]. Patients were classified as type 1, 2, or 3, according to their neurological symptoms, which were evaluated by a neurologist. In some younger patients, future neurological involvement cannot be ruled out. Moreover, in some cases, neurological disease could be due to factors other than genetic GD.

### Genetic analysis

Full *GBA *gene sequencing was not performed in all patients. Samples were first screened for N370S and L444P mutations. In some patients, N370S, L444P, D409H, G377S, N396T, 55bpdel, and N188S were the only mutations analysed. Long-chain PCR and nested PCR or PCR reactions using gene-specific primers were employed to amplify the segments of the functional *GBA *gene in patients with unknown mutant alleles. The amplification products were subjected to DNA sequencing to detect new mutations. In double heterozygous patients, the phase was established by genotyping the parents, if the material was available. To exclude the 55 bp deletion in exon 9, all patients from SGDR who were homozygous for the N370S mutation were reanalysed as previously described [15]. In addition, to identify true L444P homozygosity or rearrangements involving L444P, two fragments of 250 and 223 bp that comprise exon 9 and 10 respectively were sequenced in patients from SGDR who were apparently homozygous for L444P.

### Data analysis

Descriptive statistics were used to analyse the data in this study, according to the demographic and clinical characteristics of GD. Proportions were calculated for categorical variables (e.g., sex, genotype, ethnicity, and neurological characteristics). Summary statistics (mean, standard deviation [SD] and percentiles) were calculated for continuous measures (e.g., age). The statistical and epidemiological analyses were performed using Stata v. 10.0. The standardized incidence ratio was calculated using an indirect standardization method.

## Results

**Figure 1:**
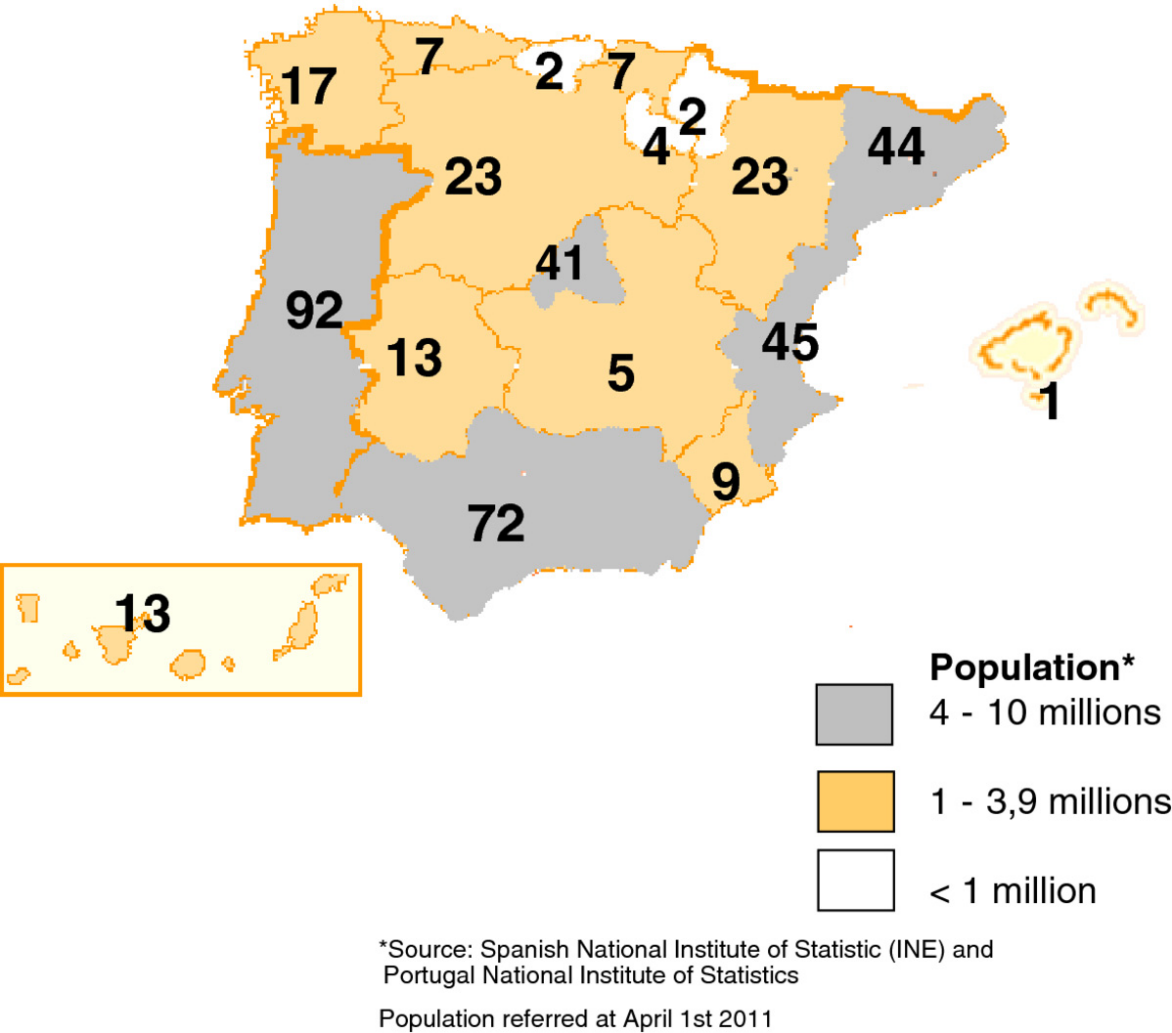
**Distribution of Gaucher Disease Patients in Iberian Peninsula and its islands**.

Of the 436 patients included in this series, 96.1% were born in the IP or the Balearic or Canary Islands (Figure 1); 92 were from Portugal (21.1%), 327 (75.0%) were of Spanish origin, and 17 (3.9%) were immigrants from Cuba (n = 1), Brazil (n = 1), Ecuador (n = 1), France (n = 2), Germany (n = 1), Guinea (n = 1), India (n = 1), Morocco (n = 2), Romania (n = 2), Syria (n = 1), Turkey (n = 1), the UK (n = 1), and Uruguay (n = 2).

Patients born in the IP were classified by diagnosis: 370 as GD type 1 (88.3%) (mean age: 40.6 ± 20.30 years, range 0.3-87); 28 as GD type 2 (6.7%) (mean age: 0.4 years, range 0-1); and 21 as GD type 3 (5.0%) (mean age: 5.9 years, range 2-17).

**Figure 2:**
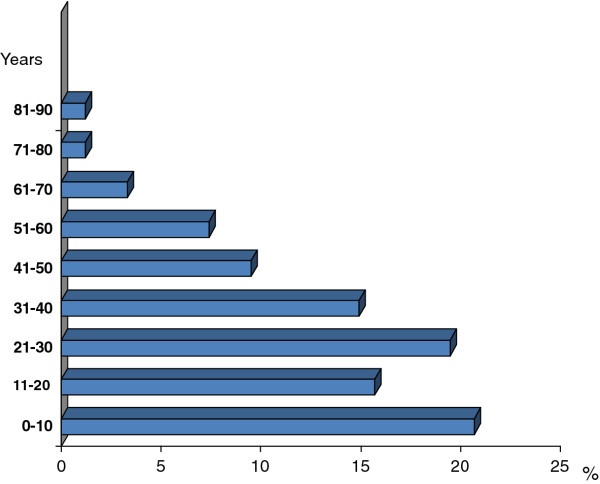
**Type 1 Gaucher Disease**. Age at diagnosis distribution.

Mean age at diagnosis among all GD patients was 26.3 ± 19.88 years (range 0-87); 34% of type 1 patients were diagnosed under 18 years old (mean age 8.8 y; range: 2.5-17 y). The majority of type 1 patients (66%) were diagnosed in adulthood (> 18 years old), mean age: 30.8 y (range 18-87 years). Figure 2 shows the distribution of age at diagnosis in type 1 GD.

No consistent data about the time between the development of the first symptoms and diagnosis were available for all patients, but we have recorded the time of first therapy. The mean time between the diagnosis and the start of therapy in type 1 patients was 18.1 years (range 0.2-48 years).

In terms of sex, 198 (47.3%) patients were female and 203 (48.4%) male, with data unavailable in 4.3% of cases. To date, 63 patients from the IP in this series have died (15%). With respect to type, 7.6% of patients with type 1, 52.4% with type 3, and 100% with type 2 have died. The cause of death for all patients with type 3 was the progression of neurological disease. The mean age of death for GD type 1 patients was 60.1 (range 33-78). The most frequent causes in this cohort were liver failure, Parkinson disease, cancer and sepsis.

The total number of inhabitants in the IP was 56,032,992 (data obtained from the Spanish National Institute of Statistics [http://www.ine.es] and the Portuguese National Institute of Statistics [http://www.ine.pt]). Therefore, the prevalence (proportion of individuals in a population suffering from GD) in the IP was 1/149,000 inhabitants.

We identified 69 alleles and 66 mutations. The allele frequency and type of *GBA *allele distributions in the IP are presented in Additional file 1: Table S1 (supplementary material). The most prevalent allelic frequencies that accounted for 75% of the total alleles were, in order of frequency: N370S (N = 406; 48.5%), L444P (N = 155; 18.5%), D409H (N = 28; 3.3%), G377S (N = 25; 3.0%), and the double mutation L444P + E326K (N = 15; 1.8%). In twenty-eight (3.3%) of the alleles, the mutation has not yet been identified, and the remaining alleles corresponded to private mutations (< 1.7% of total alleles). The distribution of *GBA *mutations by IP region is presented in Additional file 2: Figure S1 (supplementary material).

The most frequent genotypes overall were N370S/L444P (Ν = 117; 27.9%), followed by N370S/N370S (Ν = 62; 14.8%). Eighty per cent of the total GD patients (N = 335) from the IP were compound heterozygous with one N370S mutation or were homozygous for this mutation. The most frequent genotype in Portugal was N370S/N370S (N = 24; 26.1%), followed by N370S/L444P (N = 20; 21.7%). In contrast, the most frequent genotype in Spain was N370S/L444P (N = 97; 29.7%), and the second most common was N370S/N370S (N = 38; 11.6%). Nevertheless these differences were not significant (p = 0.09). In each Spanish region, the most frequent genotype was N370S/L444P while the second varied (data not shown).

In type 1 GD, the most frequent genotype was N370S/L444P (N = 117; 31.6%) followed by N370S/N370S (N = 62; 16.8%). N370S/c.84insG accounted for (N = 10) 2.7%, and other genotypes presented frequencies of less than 2.5% (Table 1). Type 2 GD patients had a broad spectrum of genotypes, and 24 (85.7%) were compound heterozygous with one mutation being L444P (Table 2). The most frequent genotypes for type 3 GD were L444P/L444P and D409H/D409H, each accounting for 28.6% (Table 3). Surprisingly, one patient with myoclonic epilepsy classified as type 3 was compound heterozygous, with one mutation being N370S. Hence, the presence of a second mutation in the N370S allele could be suspected. However, we sequenced all exons and exon-intron boundaries of *GBA *from this patient and excluded the presence of other mutations.

**Table 1:** *GBA *genotype distribution in type 1 GD

Genotype	Frequency	Percent
N370S/L444P	117	31.6
N370S/N370S	62	16.8
N370S/c.84insG	10	2.7
G377S/D409H	10	2.7
N370S/G202R	9	2.4
N370S/c.1263_1317del55	8	2.2
N370S/R120W	6	1.6
N370S/G195W	5	1.3
N370S/Rec all gene	5	1.3
N370S/RecNciI	5	1.3
N370S/T134P	5	1.3
N396T/N396T	5	1.3
G377S/G377S	4	1.1
N370S/G377S	4	1.1
N370S/N396T	4	1.1
N370S/R47X	4	1.1
N370S/RecTL	4	1.1
N370S/Y313H	4	1.1
N370S/F109V	3	< 1
N370S/L336P	3	< 1
N370S/[N188S;E326K]	3	< 1
N370S/P391L	3	< 1
N370S/R163X	3	< 1
N370S/R257X	3	< 1
N370S/R463C	3	< 1
N370S/[c.(-203)A > G;IVS4-2a > g]	3	< 1
N370S/c.500insT	3	< 1
N370S/[E326K;L444P]	2	< 1
N370S/G325W	2	< 1
N370S/IVS2+1	2	< 1
N370S/M123T	2	< 1
N370S/[RecNciI;c.1263_1317del55]	2	< 1
N370S/V15M	2	< 1
N370S/V191G	2	< 1
N370S/W(-4)X	2	< 1
N370S/Y412H	2	< 1
N370S/c.1439_1445del7	2	< 1
D409H/[N188S;E326K]	1	< 1
H311R/R359Q	1	< 1
L444P/G377S	1	< 1
L444P/L444P	1	< 1
N370S/G113E	1	< 1
N370S/IVS5+1 g > t	1	< 1
N370S/M123K	1	< 1
N370S/N188S	1	< 1
N370S/Q169X	1	< 1
N370S/R257Q	1	< 1
N370S/RecTL	4	1.1
N370S/Y313H	4	1.1
N370S/F109V	3	< 1
N370S/L336P	3	< 1
N370S/[N188S;E326K]	3	< 1
N370S/P391L	3	< 1
N370S/R163X	3	< 1
N370S/R257X	3	< 1
N370S/R463C	3	< 1
N370S/[c.(-203)A > G;IVS4-2a > g]	3	< 1
N370S/c.500insT	3	< 1
N370S/[E326K;L444P]	2	< 1
N370S/G325W	2	< 1
N370S/IVS2+1	2	< 1
N370S/M123T	2	< 1
N370S/[RecNciI;c.1263_1317del55]	2	< 1
N370S/V15M	2	< 1
N370S/V191G	2	< 1
N370S/W(-4)X	2	< 1
N370S/Y412H	2	< 1
N370S/c.1439_1445del7	2	< 1
D409H/[N188S;E326K]	1	< 1
H311R/R359Q	1	< 1
L444P/G377S	1	< 1
L444P/L444P	1	< 1
N370S/G113E	1	< 1
N370S/IVS5+1 g > t	1	< 1
N370S/M123K	1	< 1
N370S/N188S	1	< 1
N370S/Q169X	1	< 1
N370S/R257Q	1	< 1
N370S/R285C	1	< 1
N370S/R359Q	1	< 1
N370S/R359X	1	< 1
N370S/R395C	1	< 1
N370S/Rec(int2)	1	< 1
N370S/[RecTL;c.1263_1317del55]	1	< 1
N370S/S364R	1	< 1
N370S/W(-4X)	1	< 1
N370S/W184R	1	< 1
N370S/[c.(-203)A > G;P182L]	1	< 1
N370S/[c.(-203)A > G;P391L]	1	< 1
N370S/c.1097_1098delGC	1	< 1
N370S/c.1451_452delAC	1	< 1
N370S/c.1510_1512delTCT	1	< 1
N370S/c.708delC	1	< 1
N370S/c.838delT	1	< 1
R496H/R496H	1	< 1
unknown/unknown	2	< 1
[E326K;L444P]/unknown	1	< 1
N370S/unknown	22	5.9

Total	370	

**Table 2:** GBA genotype distribution in type 2 GD

Genotype	Frequency	Percent
[E326K;L444P]/R463H	3	10.7
L444P/G195E	3	10.7
L444P/c.1263_1317del55	3	10.7
D409H/R120W	2	7.1
[E326K;L444P]/[E326K;L444P]	2	7.1
L444P/L444P	2	7.1
L444P/R120W	2	7.1
[E326K;L444P]/G202R	1	3.6
[E326K;L444P]/W312R	1	3.6
G389E/unknown	1	3.6
[E326K;L444P]/L444P	1	3.6
L444P/G202R	1	3.6
L444P/I270P	1	3.6
L444P/RecNciI	1	3.6
L444P/S364R	1	3.6
L444P/c.203_204insC	1	3.6
N392I/L444P	1	3.6
V15M/G195W	1	3.6
D409H/R120W	2	7.1
[E326K;L444P]/[E326K;L444P]	2	7.1
L444P/L444P	2	7.1
L444P/R120W	2	7.1
[E326K;L444P]/G202R	1	3.6
[E326K;L444P]/W312R	1	3.6
G389E/unknown	1	3.6
[E326K;L444P]/L444P	1	3.6
L444P/G202R	1	3.6
L444P/I270P	1	3.6
L444P/RecNciI	1	3.6
L444P/S364R	1	3.6
L444P/c.203_204insC	1	3.6
N392I/L444P	1	3.6
V15M/G195W	1	3.6

Total	28	

**Table 3:** GBA genotype distribution in type 3 GD

Genotype	Frequency	Percent
D409H/D409H	6	28.6
L444P/L444P	6	28.6
L444P/D409H	2	9.5
[E326K;N188S]/L444P	2	9.5
[E326K;L444P]/D409H	1	4.8
[E326K;L444P]/P182L	1	4.8
G377S/G195W	1	4.8
N370S/G195W	1	4.8
R463C/G377S	1	4.8

Total	21	

Eight patients homozygous for L444P, with no neurological involvement at diagnosis were reclassified: two as type 2 (0.5-1 years) and six as type 3 (range 4-41 years).

Several patients described in our series had interesting phenotypes. G377S/G195W and G377S/R463C were found to be associated with type 3 GD, as also were the homozygous for D409H. All patients with a genotype homozygous for D409H developed cardiovascular and neurological signs in the second decade of life (mean age: 14.7 years; range 10-17).

However, neither patients with the G377S/D409H genotype nor those who were G377S homozygous developed neurological symptoms. We found two families with the G377S/D409H genotype (10 patients), with a mean age of 57.1 years (range: 50-64 years) without neurological manifestations. Two patients, one homozygous for L444P (11 years old) and the other one (30 years old) with genotype D409H/N188S;E326K, were classified as type 1 because no neurological signs of disorder were observed.

For the analysis of clinical data, we considered only the 357 patients for whom we had full information at diagnosis. The mean SSI in type 1 GD was 7.5 ± 3.59 (range 1-25); two hundred and fifty-nine patients (72.7%) were classified as having a mild form, 91 (25.5%) moderate, and 7 (1.8%) severe, according to the Zimran score [17].

A total of 58 (16.2%) patients were asymptomatic. Most of this group were diagnosed by screening the family members of an affected GD. The asymptomatic patient genotypes were homozygous for N370S, compound heterozygous with one mutation being N370S, and patients with the D409H/G377S genotype. It is remarkable that 62 (17.4%) patients had been splenectomized and that in 40 (64.5%) of these cases the procedure was performed before enzyme replacement therapy (ERT) was available, this observation is the consequence of adequate therapy. In the remaining 22 cases, the spleen removal was performed in different circumstances before the diagnosis of GD. A total of 315 (88.4%) non-splenectomized patients at diagnosis had spleen enlargement. Only 5 type 1 patients had documented pulmonary hypertension and all of them were previously splenectomized.

The liver was enlarged (1.25 times the predicted normal) in 243 (68.0%) patients at diagnosis, 48 (77.4%) splenectomized and 171 (58.1%) non-splenectomized.

Without taking into account the Erlenmeyer flask deformity, 211 (62%) of all patients had skeletal involvement; 42 (67.7%) splenectomized and 132 (44.7%) non-splenectomized patients had bone disease (ranging from painful crises to fractures); and orthopedic surgery had been required in 98 (27.5%) cases.

Anemia in female patients (N = 169) (hemoglobin < 12 g/L) was present in 95 (56.2%) cases, and 107 (56.9%) men had hemoglobin levels less than 13 g/L. A total of 198 (55.5%) GD patients at diagnosis had thrombocytopenia (platelet counts < 100 × 10^9^/L), and 29 (14.4%) had severe thrombocytopenia (platelet counts < 50 × 10^9^/L). Leucopenia (leukocytes < 4 × 10^9^/L) was present in 106 (29.7%) patients. Ferritin levels were increased (> 500 μg/dL) in 165 (46.1%) patients; 322 (90.2%) had hypergammaglobulinemia (> 1.5 g/dL); and 61 adult patients (> 18 years old) (17.1%) had monoclonal gammopathy of undetermined significance (MGUS). A total of nine patients had documented cancer: 3 multiple myeloma, 2 lymphoproliferative disorder, 1 malignant melanoma, 1 colon carcinoma, 1 liver carcinoma and 1 patient with meningioma and gastric carcinoma.

According to the available data, a total of 299 patients (83.8%) had received some kind of therapy: 246 patients (82.3%) had received ERT for a mean of 10.2 ± 3.8 years (range: 1-17) and mean doses of 36 ± 12 IU/kg every 2 weeks (range: 120-10 IU/kg every two weeks). In 53 patients (17.7%), oral therapy with miglustat (100 mg, t.i.d.) had been administered for a mean of 2.1 ± 1.9 years (range: 1-6).

## Discussion

The present series appears to comprise the vast majority of IP patients known to have had GD since 1970. To date, few studies have described the characteristics of all patients diagnosed with GD in a large non-Jewish population [20,21]. This study is the first that presents GD data from the majority of patients diagnosed in Spain and Portugal.

Our data indicate that the prevalence of GD in the IP was 1:149,000. This prevalence is similar to that calculated for other EU populations, with the exception of Ashkenazi Jews [22-24]. It is likely that there are undiagnosed patients. In this regard, it is noteworthy that in a previous study of IP patients with GD, the number of homozygotes for the N370S mutation calculated from the general population frequency was higher than the number of patients diagnosed [23-25]. Also of note is the large influx of immigrants into the IP in recent years, which is reflected in the finding that 3.9% of all patients were foreign. The distribution of patients by geographical area is in line with the number of inhabitants, ranging from 0.1 to 1.5:10^5^. An exception is the Balearic Islands, where only one patient was found. Given that the population of these islands is 1,100,000 inhabitants, a minimum of 7 patients should have been identified. We do not know whether this variability in the prevalence of GD between different regions of the IP is due to asymptomatic patients or misdiagnosis. The distribution by sex was practically the same, as expected, which in part reflects the fact that the disease is autosomal recessive [1].

As in other series, type 1 was the most frequent (88.3% of total cases). Some patients diagnosed previously as type 1 were reclassified as type 3 due to the late onset of neurological symptoms. We want to emphasize the importance of periodically performing a careful neurological examination of all patients, particularly those who have genotypes considered at high risk for neurological involvement [5].

The identification of GD can be complex and lengthy; while acute or rapidly progressing cases may be identified more quickly, the disease often progresses slowly, and signs and symptoms may be subtle and easily overlooked. The age at diagnosis in type 1 GD in this series was mainly adulthood (mean age 28.7 years). This observation contrasts with other lysosomal storage diseases that are usually diagnosed in childhood. The lack of early diagnosis of type 1 GD is probably related to severity, as 72.7% of all patients had mild forms of the disease according to the SSI. Mean age at diagnosis in patients from the IP was similar to that found in patients with the N370S/N370S genotype included in the International Gaucher Registry (the ICGG Registry) [26]. If we compare the age at diagnosis of patients in the IP with the Spanish Gaucher Disease Registry data published 10 years ago, we can see a shift toward diagnosis at younger ages [16]. This shift may be due to a greater awareness of the disease in recent years and the availability of diagnosis. On the other hand, there is not a rigid relationship in type 1 patients between genotype and age at diagnosis. Affected siblings with an identical *GBA *genotype frequently developed different onset of symptoms and clinical manifestations.

In relation to spleen removal, patients who had undergone splenectomy were more common among older GD patients. We note that the proportion of splenectomized patients was lower than that observed in a similar series published 10 years ago [16]. The difference may result from the availability of enzyme treatment therapy and is the logical consequence of adequate therapy, which has reduced the practice of splenectomy.

More than half of the patients had thrombocytopenia (platelet count < 100 × 10^9^/L), and 14.4% had severe thrombocytopenia (platelet count < 50 × 10^9^/L). Similar data for platelet counts in GD patients were reported from the ICGG Registry: 15% demonstrated severe thrombocytopenia with platelet counts less than 60 × 10^9^/L; 45% had moderate thrombocytopenia (platelets > 60- < 120 × 10^9^/L) at diagnosis. However, the percentage of patients with anemia in the IP (56%) was higher than that reported by the ICGG Registry, which showed that 36% of registered patients were anemic at diagnosis. This may result from the different criteria used to consider a patient anemic, depending on hemoglobin levels. We used the WHO criteria, whilst data from the ICGG Registry were based on hemoglobin concentrations for men and women of < 12 and < 11 g/dL, respectively [27,28]. Most cases of anemia in GD patients are thought to be related to increased red blood cell destruction in the spleen [29]. Hepatomegaly was found in 68% of patients. The effect of splenectomy on liver enlargement was considerable, with 58.1% of patients in the non-splenectomized cohort presenting with hepatomegaly, compared with 77.4% in the asplenic cohort (p < 0.01).

The association of GD with polyclonal and monoclonal gammopathies has previously been reported [30,31]. A high percentage of GD patients in this series had hypergammaglobulinemia and MGUS, as well as increased ferritin levels. It has been postulated that progressive glucocerebroside accumulation may cause chronic stimulation of the immune system and consequent lymphoproliferation [32]. Ferritin release from Gaucher cells has been identified as involved in reducing T-cell function and immunoglobulin M release from B-cells [33].

As in other series, we found that bone involvement was one of the most disabling aspects for patients with type 1 GD: 62% of all patients had some type of bone involvement, including bone crises and fractures. This condition was more frequent in asplenic patients than in patients with spleens, suggesting that splenectomy worsened bone disease. Similar results were observed in patients included in the ICGG Registry [26]. Furthermore, splenectomized patients had larger livers and more frequently reported a history of bone pain, bone crises, and severe radiological evidence of bone disease than non-splenectomized patients. A total of 27.5% of patients needed to undergo an orthopedic procedure in spite of the ERT. This indicates that bone disease is an unresolved problem in a high percentage of patients.

Analysis of the mutations in GD is an interesting way of gaining knowledge about the geographical and ethnic origins of the disease. A total of 69 alleles and 66 different mutations have been identified in the IP. We included several variants in the analysis because they produce a more severe phenotype in combination with another mutation. This is the case of the variant E326K associated with the L444P and N188S mutations. The double-mutant allele [L444P;E326K] was found to further decrease residual enzyme activity and to be associated with a more severe phenotype than expected from the single L444P allele. The same was found for the polymorphism c.(-203)A > G[34,35].

The most frequent mutation in individuals with GD in the IP was N370S, followed by L444P. The two mutations together accounted for 67% of total alleles. This finding has important implications for genetic diagnosis. Initially, the analysis can focus on these two mutations. If both alleles are identified, *GBA *gene sequencing is not necessary. However, the number of different mutations in the remaining 33% of alleles identified in the *GBA *gene in the IP is high, making genetic characterization more complex. Interestingly, ten patients had c.84insG. This insertion had been considered to occur only in Ashkenazi Jews.

Although there is no strict relationship between genotype and phenotype, the fact that most patients are compound heterozygous with one mutation being N370S, which is considered a low-severity mutation, could be a plausible explanation for the high percentage of patients with mild and moderate forms of the disease (72.7% and 25.5%, respectively).

The most frequent mutation among type 1 patients was N370S, which accounted for 54.7% of total alleles. However, in type 3 patients, there was one compound heterozygous individual, with one mutation being N370S (genotype N370S/G195W). It is important to point out that we do not know if the cause of neurological impairment in this case was the *GBA *gene or another gene, a gene-gene interaction, or a gene-environment interaction. L444P was the most frequent mutation in patients with type 2, as well as among type 3 patients, where it accounted for 33.9% and 38.1% of total alleles, respectively. It is interesting to note that homozygosity for L444P was found in two patients classified as type 2 and in six as type 3, and homozygosity for [E326K; L444P] occurred only in one type 2 GD patient.

Two mutations, D409H and G377S, represented 6.3% of total alleles. Both mutations in homozygosity or in heterozygosity with another mutation have been associated with a broad spectrum of GD phenotypes. This is the case for the D409H mutation in which homozygous patients present a rare form of type 3 GD characterized by heart valve calcification [36]. In contrast, unlike a report from Croatia, we did not observe heart valve disease in the four patients homozygous for G377S [37]. This finding further proves that patients with the same genotype can have different phenotypes, which emphasizes the influence of other genetic and/or environmental factors.

Surprisingly, certain genotypes were not associated with neurological involvement. This is the case of GD patients carrying the fourth most frequent mutation, G377S, which in homozygosity (G377S/G377S) or in heterozygosity with the D409H mutation (G377S/D409H) or even with the L444P mutation (G377S/L444P) resulted in only mild or moderate forms of the disease, with no neurological involvement. However, this mutation in compound heterozygosity with two missense mutations, G195W or R463C (G377S/G195W or G377S/R463C genotypes), was found in patients who developed neurological symptoms during childhood (type 3 GD patients). Similar observations were reported in patients from Brazil, who were compound heterozygous with one mutation being G377S [38]. Another interesting observation was the combination of the double-mutant allele [E323K;N188S] with two mutations that are considered severe: D409H and L444P. The genotype L444P/[E323K;N188S] was found in patients with GD type 2/3, and the genotype D409H/[E326K;N188S] was present in GD type 1/3 patients. These findings indicate that several mutations cannot be unambiguously classified as mild and suggest an allele-dose effect for them.

Before the problem of the imiglucerase shortage (June 2009 to the end of 2010), most patients were under ERT, and only a smaller percentage were treated with miglustat. It is important to note that the number of patients treated with miglustat was less than the ERT value, because the indication for treatment is specific to individual patients.

In conclusion, we found a large heterogeneity of *GBA *gene mutations among IP patients and the clinical presentation of GD patients, and little relationship between genotype and phenotype. The prevalence of the N370S mutation in Spanish GD patients is one of the highest in Europe, and that of L444P is one of the lowest worldwide. These findings contrast with observations in closed populations in which the "founder" effect is evident. In such populations, the number of mutations giving rise to GD is small, which facilitates a rapid genetic diagnosis. The information presented here adds to our understanding of the clinical spectrum of GD, highlights the genetic and phenotypic heterogeneities and emphasizes the need for caution in making generalizations about GD across demographic groups.

## Competing interests

The authors declare that they have no competing interests.

## Authors' contributions

Study conception and design: Dr. Giraldo, Dr. Pocovi, Dr Sa Miranda, Dr Grinberg, Dr Vilageliu; Analysis and interpretation of data: Dr. Alfonso, Dr. Irún, Dr. Gort, Dr. Chabas; Drafting the article or revising it critically for important intellectual content: Dr. Giraldo, Dr. Pocovi, Dr Chabas, Dr Grinberg, Dr Vilageliu.

## Disclosures

Conflict of interest: none declared.

## Supplementary Material

Additional file 1 **Table S1(Supplementary material *GBA *allele frequencies in GD patients born in IP (Spain and Portugal)**. Click here for file

Additional file 2 **Figure S1 Allelic distribution of *GBA *mutations in Iberian Peninsula and its islands**. Click here for file
