# Supplementary material for: Mapping the genetic and clinical characteristics of Gaucher disease in the Iberian Peninsula
Source: Orphanet J Rare Dis. 2012 Mar 19;7:17. doi: 10.1186/1750-1172-7-17 (PMC3349595; doi:10.1186/1750-1172-7-17)
Supplement: Additional file 1 — Table S1(Supplementary material GBA allele frequencies in GD patients born in IP (Spain and Portugal). [file 1750-1172-7-17-S1.DOCX]

**Table 1S (Supplementary material):** *GBA* allele frequencies in GD patients born in IP (Spain and Portugal)

Allele Frequency Percent

-----------------------------------------

N370S 406 48.5

L444P 155 18.5

D409H 28 3.3

G377S 25 3.0

[E326K;L444P] 15 1.8

N396T 14 1.7

G202R 11 1.3

c.1263_1317del55 11 1.3

R120W 10 1.2

c.84insG 10 1.2

G195W 8 1.0

[E326K;N188S] 6 <1

RecNciI 6 <1

Rec all gene 5 <1

T134P 5 <1

R463C 4 <1

R47X 4 <1

RecTL 4 <1

Y313H 4 <1

F109V 3 <1

G195E 3 <1

L336P 3 <1

P391L 3 <1

R163X 3 <1

R257X 3 <1

R463H 3 <1

V15M 3 <1

W(-4)X 3 <1

[c.(-203)A>G;IVS4-2a>g] 3 <1

c.500insT 3 <1

G325W 2 <1

IVS2+1 2 <1

M123T 2 <1

R359Q 2 <1

R496H 2 <1 [RecNciI;c.1263_1317del55] 2 <1

S364R 2 <1

V191G 2 <1

Y412H 2 <1

c.1439_1445del7 2 <1

G113E 1 <1

G389E 1 <1

H311R 1 <1

I270P 1 <1

IVS5+1g>t 1 <1

M123K 1 <1

N188S 1 <1

N392I 1 <1

P182L 1 <1

Q169X 1 <1

R257Q 1 <1

R285C 1 <1

R359X 1 <1

R395C 1 <1

Rec(int2) 1 <1

[RecTL;c.1263_1317del55] 1 <1

W184R 1 <1

W312R 1 <1

[c.(-203)A>G;P182L] 1 <1

[c.(-203)A>G;P391L] 1 <1

c.1097_1098delGC 1 <1

c.1451_1452delAC 1 <1

c.1510_1512delTCT 1 <1

c.203_204insC 1 <1

c.708delC 1 <1

c.838delT 1 <1

unknown 28 3.3

----------------------------------------------------

Total 838
