# Supplementary figures and images for: Mapping the genetic and clinical characteristics of Gaucher disease in the Iberian Peninsula
Source: Orphanet J Rare Dis. 2012 Mar 19;7:17. doi: 10.1186/1750-1172-7-17 (PMC3349595; doi:10.1186/1750-1172-7-17)

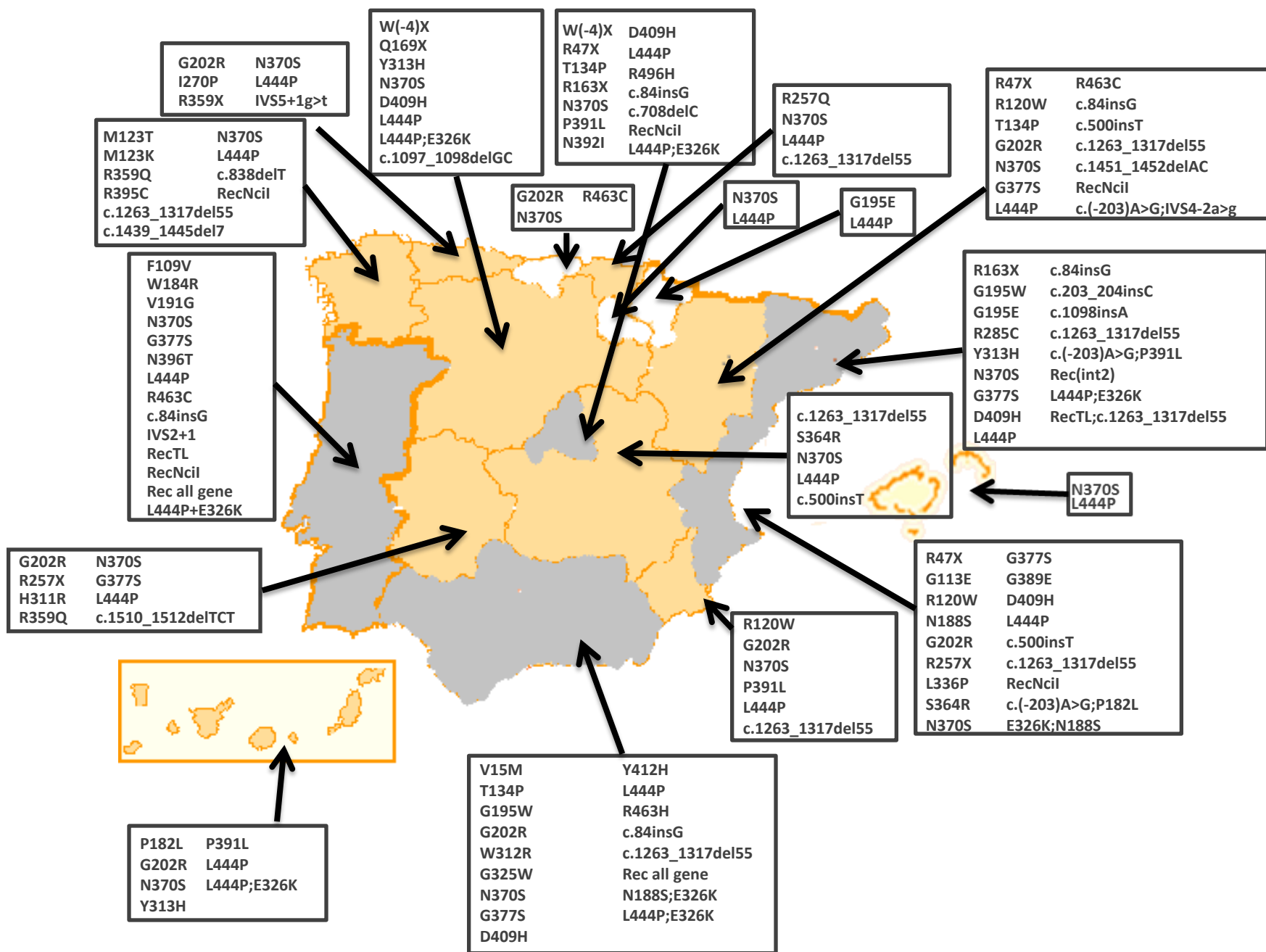

Supplement: Additional file 2 — Figure S1 Allelic distribution of GBA mutations in Iberian Peninsula and its islands. [file 1750-1172-7-17-S2.PDF]
